# Institutional maternal and perinatal deaths: a review of 40 low and middle income countries

**DOI:** 10.1186/s12884-017-1479-1

**Published:** 2017-09-07

**Authors:** Patricia E. Bailey, Wasihun Andualem, Michel Brun, Lynn Freedman, Sourou Gbangbade, Malick Kante, Emily Keyes, Edwin Libamba, Allisyn C. Moran, Halima Mouniri, Dahada Ould el Joud, Kavita Singh

**Affiliations:** 1 Global Health Programs, FHI 360, 359 Blackwell Street, Durham, NC 27701 USA; 2 Averting Maternal Death & Disability, Columbia University, New York, NY USA; 3 Independent consultant, Addis Ababa, Ethiopia; 4 UNFPA, New York, NY USA; 5 Independent consultant, Cotonou, Benin; 6 Independent consultant, Lilongwe, Malawi; 7 U.S. Agency for International Development, Washington, DC USA; 8 Independent consultant, Rabat, Morocco; 9 Independent consultant, Nouakchott, Mauritania; 10 MEASURE Evaluation, Carolina Population Center, University of North Carolina at Chapel Hill, Chapel Hill, NC 27516 USA; 11 Department of Maternal and Child Health, Gillings School of Global Public Health, University of North Carolina at Chapel Hill, Chapel Hill, NC 27599 USA

**Keywords:** Cause of maternal death, Direct and indirect deaths, Cause specific case fatality rate, Stillbirth rate, Early neonatal death rate, Perinatal mortality

## Abstract

**Background:**

Understanding the magnitude and clinical causes of maternal and perinatal mortality are basic requirements for positive change. Facility-based information offers a contextualized resource for clinical and organizational quality improvement. We describe the magnitude of institutional maternal mortality, causes of death and cause-specific case fatality rates, as well as stillbirth and pre-discharge neonatal death rates.

**Methods:**

This paper draws on secondary data from 40 low and middle income countries that conducted emergency obstetric and newborn care assessments over the last 10 years. We reviewed 6.5 million deliveries, surveyed in 15,411 facilities. Most of the data were extracted from reports and aggregated with excel.

**Results:**

Hemorrhage and hypertensive diseases contributed to about one third of institutional maternal deaths and indirect causes contributed another third (given the overrepresentation of sub-Saharan African countries with large proportions of indirect causes). The most lethal obstetric complication, across all regions, was ruptured uterus, followed by sepsis in Latin America and the Caribbean and sub-Saharan Africa. Stillbirth rates exceeded pre-discharge neonatal death rates in nearly all countries, possibly because women and their newborns were discharged soon after birth.

**Conclusions:**

To a large extent, facility-based findings mirror what population-based systematic reviews have also documented. As coverage of a skilled attendant at birth increases, proportionally more deaths will occur in facilities, making improvements in record-keeping and health management information systems, especially for stillbirths and early neonatal deaths, all the more critical.

## Background

Post Millennium Development Goal global action agendas such as the Sustainable Development Goals (SDGs), Every Newborn Action Plan (ENAP) and Ending Preventable Maternal Mortality continue to measure global progress to reduce the maternal mortality ratio (MMR), the neonatal mortality rate, and now (under ENAP guidance) the stillbirth rate [1–3]. Understanding the magnitude and clinical causes of maternal and perinatal mortality are basic requirements for policy setting, program design, innovation testing, and the implementation of evidence-based interventions. Understanding maternal and newborn outcomes captured at health facilities presents an opportunity for health care staff and decision-makers to reflect on what they could do better.

High quality data on how many maternal and newborn deaths occur and their causes are notoriously difficult to come by and global estimates come from complex models based on multiple sources: vital registration data, confidential enquiries, large household surveys, reproductive-age mortality studies, research reports, surveillance data, and verbal autopsies [4–8]. Over the last few decades the Maternal Mortality Estimation Inter-Agency Group produced a series of maternal mortality estimates [7, 9, 10], the Global Burden of Disease Studies contributed important systematic analyses of trends, projections, and causes of maternal and child mortality [6, 11, 12], while the World Health Organization (WHO) produced two systematic analyses of the global causes of maternal death [13, 14]. The authors of these comprehensive systematic reviews shy away from using routine health facility data because of inherent selection bias. However, in the 2003–2009 WHO systematic analysis, the authors consulted health facility data when the institutional delivery rate in that country was 50% or greater during the period reviewed [14].

Globally, the coverage of skilled attendant at birth increased from 59% in 1990 to 71% in 2015 [15]. More countries are adopting a 100% institutional delivery policy and institutional delivery rates are rising. This shift means that proportionately more individuals with peripartum and perinatal complications will access treatment, and mortality events, when they happen, are more likely to occur in facilities than at home. In low and middle income countries facility-based maternal and perinatal mortality figures do not yet substitute population-derived estimates as they reflect only those women and newborns who succeed in accessing facility-based care. Facility-based events are highly specific to local contextualized conditions, and thus, are well-suited to inform local policy makers, clinicians and programs to target specific health system strengthening efforts. Most importantly, they can be used to improve service quality. As the SDGs bring a renewed global focus on improving the quality of routine health management information systems, through the Health Data Collaborative and other initiatives, and as they include the use of new technologies, data quality and availability will increase and the cost of collecting data will decrease.

This paper reviews data from up to 40 low and middle income countries and describes the magnitude of institutional maternal mortality, causes of maternal death and cause-specific case fatality rates, as well as institutional stillbirth and early or pre-discharge neonatal death rates, in most cases, at the national level. This analysis draws on reports produced over the last decade.

## Methods

This secondary data analysis is based on a review of cross-sectional health facility surveys known as emergency obstetric and newborn care (EmONC) assessments, which focus on routine intrapartum care for women and their newborns as well as more complicated births. These assessments have been driven by the United Nations Fund for Population (UNFPA), the United Nations Children’s Fund (UNICEF), the WHO, and the Averting Maternal Death and Disability (AMDD) program at Columbia University. The methods have been described elsewhere, but a summary follows [16, 17].

### Sampling

Most EmONC assessments were national in scope and targeted facilities providing childbirth services. As a rule, all hospitals were selected, and if a “census” of childbirth sites was not possible, hospitals were supplemented by either a random sample of mid-level facilities (health centers, clinics), or a “restricted census” of higher volume mid-level facilities that attended more than a specified number of monthly deliveries. Usually, both private and public sector facilities were included. Table 1 shows the number of hospitals and other facilities surveyed in each country and the population size covered by the facilities visited.

**Table 1:** EmONC assessment characteristics and facility-based rates and ratios (40 countries)

Region, country and year of data collection	Population covered	No. of health facilities providing delivery services	No. of hospitals	No. of other facilities	Institu-tional deliveries (12 mo.s)	Expected births (12 mo.s)	Institu-tional delivery rate	Percentage of deliveries with obstetric complica-tions^e^	Institutional maternal deaths^f^ (12 mo.s)	Institu-tional MMR	MMR (2015)
LAC											
Ecuador 2006^a^	690,049	9	9	0	8863	NR	NR	30.0%	7	79	64
Guyana 2010	751,223	51	32	19	12,803	14,724	87.0%	7.1%	19	148	229
Haiti 2009	9,761,927	120	59	61	65,731	294,034	22.4%	20.8%	170	259	359
Nicaragua 2006	5,626,493	167	20	147	94,136	176,409	53.4%	7.2%	41	44	150
Panama 2007	2078,446	19	11	8	30,811	52,310	58.9%	7.5%	21	68	94
Western Africa											
Benin 2010	8,497,827	417	34	383	203,412	349,856	58.1%	11.8%	483	237	405
Burkina Faso 2010	15,224,781	1626	61	1565	499,753	697,295	71.7%	9.6%	676	135	371
Cote d’Ivoire 2010	21,693,185	1419	86	1333	342,936	790,776	43.4%	13.7%	1416	413	645
Gambia 2012	1,839,447	98	8	90	51,518	69,899	73.7%	10.7%	169	328	706
Ghana 2010	24,232,431	1268	285	983	434,508	751,205	57.8%	20.1%	840	193	319
Guinea 2011	11,211,223	502	49	453	141,724	438,293	32.3%	8.3%	459	324	679
Liberia 2010	3,709,850	304	27	277	46,841	159,524	29.4%	11.7%	335	715	725
Mauritania 2011	3,297,000	254	18	236	83,409	159,533	52.3%	5.0%	132	158	602
Niger 2010	15,203,822	503	36	467	152,415	677,352	22.5%	11.7%	1165	970	553
Senegal 2013	12,873,601	560	29	531	237,494	496,921	47.8%	5.0%	1020	429	315
Sierra Leone 2008^a^	5,532,000	145	38	107	25,447	254,472	10.0%	11.4%	212	833	1360
Togo 2012	6,191,155	864	46	818	133,119	202,451	65.8%	10.7%	225	169	368
Eastern Africa											
Burundi 2010	8,246,878	274	48	226	231,293	323,278	71.5%	4.5%	220	95	712
Djibouti 2005	636,540	16	7	9	11,636	22,289	52.2%	24.4%	22	189	229
Eritrea 2008^b^	3,543,578	118	18	100	25,000	NR	26.0%	22.3%	41	164	501
Ethiopia 2008–9	73,918,505	751	112	639	174,561	2,638,891	6.6%	13.9%	685	392	353
Madagascar 2009	19,378,009	294	147	147	118,774	647,226	18.4%	17.4%	357	301	353
Malawi 2014^c^	15,805,239	365	87	278	476,272	790,262	60.3%	8.4%	586	123	634
Mozambique 2012^g^	23,569,908	947	56	891	647,944	895,656	72.3%	4.3%	1840	284	489
Rwanda 2007^a,h^	8,934,215	407	39	368	207,738	384,171	54.1%	3.4%	294	142	290
South Sudan 2013	10,864,357	407	63	344	52,842	456,303	11.6%	10.2%	497	941	789
Zanzibar 2012^a^	1,460,987	79	43	36	27,102	54,057	50.1%	9.3%	62	229	NR
Zambia 2014-15^c^	15,023,315	384	118	266	475,646	644,500	73.8%	5.0%	759	160	224
Central Africa											
Angola 2007	18,176,685	400	84	316	248,187	872,481	28.4%	12.5%	1410	568	477
Cameroon 2010	15,544,387	607	123	484	282,486	615,558	45.9%	11.4%	744	263	596
Chad 2011	11,679,974	139	57	82	49,202	520,927	9.4%	9.3%	1048	2130	856
Congo 2012	4,085,422	240	32	208	85,038	170,362	49.9%	5.1%	246	289	442
Dem Rep Congo 2011	15,421,731	266	69	197	158,546	616,869	25.7%	10.8%	282	178	693
São Tomé & P 2013	178,739	6	1	5	5455	6166	88.5%	3.2%	6	110	156
Southern Africa											
Lesotho 2015	1,954,906	160	22	138	43,165	58,197	74.2%	8.1%	66	153	487
Namibia 2005	2,028,238	100	41	59	44,592	62,875	70.9%	6.7%	57	128	265
Asia											
Afghanistan 2009	23,500,000	78	69	9	192,627	993,840	19.4%	12.9%	258	134	396
Bangladesh 2012^d^	43,667,450	846	234	612	253,728	NR	NR	17.3%	377	149	176
Cambodia 2014	13,388,910	180	91	89	119,931	382,830	31.3%	8.2%	57	48	161
Mongolia 2009	1,139,462	21	21	0	31,012	27,448	113.0%	28.3%	10	32	44

*NR* not reported, *LAC* Latin America and the Caribbean, *MMR* maternal mortality ratio, *mo* months, *MMR* (2015) see reference [7]

^a^Ecuador, Democratic Republic of Congo, Rwanda, São Tomé and Príncipe, Sierra Leone and Zanzibar reported only direct maternal deaths

^b^Eritrea: 25,000 deliveries are live births, based on 2006 data, not EmONC assessment; institutional delivery rate also not based on EmONC assessment

^c^Malawi and Zambia: deaths and deliveries weighted; in Malawi unweighted deliveries = 367,738 and unwt deaths = 557; in Zambia, unweighted deliveries = 254,790 and unwt deaths = 673

^d^Bangladesh health facilities that performed cesareans were considered hospitals; if not, considered “other”. Sample included 24 districts

^e^Complications included only major direct obstetric complications (hemorrhage, severe pre-eclampsia/eclampsia, sepsis, prolonged/obstructed labor, severe abortion complications, ruptured uterus, ectopic pregnancy)

^f^Maternal deaths include all maternal deaths (direct, indirect and unknown causes)

^g^Mozambique: Based on 3 months of data, multiplied by 4 to show 12 months, for consistency across countries

^h^Rwanda: Based on 6 mo.s of data for facility births, complications and deaths, multiplied by 2 to show 12 months of data, for consistency across countries

### Primary data collection instruments

In each country, a core team adapted a set of standardized instruments that covered the availability and status of infrastructure, human resources, drugs, equipment, and supplies, and service statistics, in addition to a provider interview and chart reviews [18]. Most relevant to this paper was the 12-month retrospective summary of service statistics that included the number of deliveries, women experiencing obstetric and non-obstetric complications by type of complication, maternal deaths by cause, and birth outcomes. Data collectors extracted data from logbooks in labor and delivery wards, maternity wards, operating theatres, and newborn care units in each facility. When any doubt or clarification was required, data collectors turned to the staff on duty.

Definitions of causes of maternal death were informed by the international statistical classification of diseases and related health problems, 10th edition (ICD-10) and its application to deaths during pregnancy, childbirth and the puerperium (ICD-MM). Obstetric complications were elaborated upon to distinguish between antepartum and postpartum hemorrhage and retained placenta. Prolonged and obstructed labor were included, sometimes joined as one category. Ruptured uterus and ectopic pregnancy along with postpartum sepsis, severe pre-eclampsia and eclampsia were the final “major direct complications” listed on the instrument. Indirect complications included malaria, HIV/AIDS, severe anemia, and less commonly, hepatitis and diabetes. In each case, the form included a category for “other” direct complications and “other” indirect complications. Causes of death mirrored the listing of complications. Finally, space permitted the reporting of unspecified/unknown causes of maternal death. For the 12-month summary of maternal deaths, the data collectors were guided by the primary sources they located on the wards or with the staff. Where maternal death audits or reviews took place, those records were also accessed, but generally no subsequent recoding was performed.

The 12-month retrospective compilation of service statistics was also designed to test the intrapartum and early neonatal death rate as an indicator of intrapartum care quality [19]. Data extraction from maternity or delivery registers captured the number of antepartum (macerated) and intrapartum (fresh) stillbirths, defined by 28 weeks of gestation or more. Intrapartum stillbirths and live births were divided between those weighing above and below 2500 g. Early neonatal deaths were defined as those occurring before discharge or within the first 24 h, whichever came first. Countries varied widely as to level of detail captured, and thus, categories were added for unspecified stillbirths and birth weights when the timing of death or birth weight was not recorded, and for live births and early neonatal deaths when birth weight was not recorded.

These categories for maternal and newborn outcomes were standardized across countries. Data collectors were trained to use a manual with the same definitions for each obstetric complication, type of stillbirth and early neonatal death.

### Secondary analysis

EmONC assessment final reports were the source of most of the data compiled in this paper; we had access to primary data in nine countries, but only in two or three situations did we access those data. Because reporting was largely driven by country interests, not all reports contained the same information nor was it presented in a standardized fashion. Consequently, the number of countries in each table differs. For example, some countries did not report the major obstetric complications by type of complication, making it impossible to calculate cause specific case fatality rates. One report candidly reported that the number of maternal deaths was grossly underreported and was not included. Other countries presented the intrapartum and pre-discharge neonatal death rate as recommended, restricting the numerator and denominator to babies weighing 2500 g or more, but they failed to report *all* stillbirths, nor did they report the number for which birth weight or stillbirth timing was unspecified; these data were not included in the paper. A small number of countries reported only direct maternal deaths, omitting the number of unspecified/unknown maternal deaths or indirect deaths; these reports were retained. Some countries distinguished between antepartum hemorrhage and postpartum hemorrhage, while others reported the two together.

About 10 of the 40 countries had conducted more than one EmONC assessment. In all cases, we extracted information from the most recent report except for Ethiopia, whose final report for their most recent assessment was not yet available.

Based on numbers drawn from the reports, we calculated the percentage of deliveries with obstetric complications and the institutional maternal mortality ratio, using 100,000 deliveries rather than live births since some countries only counted deliveries. We also calculated any regional aggregations, newborn mortality rates, and the ratio of stillbirths to early neonatal deaths. The case fatality rate was calculated by dividing the number of maternal deaths due to a specific complication by the number of complications treated. The stillbirth rate was estimated by dividing the total number of stillbirths by all deliveries (multiplied by 1000); the pre-discharge neonatal mortality rate was similar but we removed the deliveries resulting in a stillbirth from the denominator.

### Data collection and management

Ministries of Health provided oversight to all EmONC assessments and were usually supported by a technical steering committee. Public or private research institutions, universities, or central statistical offices were the most common implementing bodies for the assessments. Data collection teams usually consisted of four data collectors, generally having a health background. Data collectors participated in a weeklong training that included a review of each questionnaire, role plays, and exercises to familiarize themselves with the questionnaires and the data collectors’ manual. Each training included a one-day field activity in local hospitals and health centers where teams completed the questionnaires under supervision. Generally, quality assurance teams closely monitored the first week or two of field activities. Teams usually required one to two days to complete a hospital and half a day to complete a health center.

Data collection was paper-based for all countries but one, and data entry performed with CSPro. Report analyses were produced with statistical software such as STATA, SPSS or sometimes excel. When mid-level facilities were sampled, the data were weighted based on selection probability. Weighting is required to account for the non-uniform selection probabilities that would affect how data from selected facilities represent all facilities, including those not selected.

Technical support was provided by consultants to the AMDD program. Countries varied by the intensity of support – from no direct AMDD support (Ecuador, Panama, Cote d’Ivoire, Eritrea), to minimal remote support (Mongolia, Cambodia, Afghanistan), to most countries with more intensive support. UNFPA and UNICEF were the predominant supporters for EmONC assessments but bilateral partners and foundations also played important roles depending on the country.

### Ethical concerns

Names of women or other identifying information were never included in the primary data collection. Countries followed the guidance of their ministries of health and when required, approval of the protocols and data collection instruments from local institutional review boards was obtained. No additional approval was sought for this paper since the primary source of the data were reports in the public domain.

## Results

Up to 40 country reports (including Zanzibar) provided the number of maternal deaths that took place within health care institutions, 31 from sub-Saharan Africa, and the remaining nine from Latin America and the Caribbean and Asia (Table 1). The scope of EmONC assessments ranged from all nine hospitals in the province of Azuay, Ecuador to 1626 facilities in Burkina Faso, inclusive of all facilities with at least one delivery in the past 12 months. The total number of facilities (15,411) registered 6.5 million deliveries and 17,314 maternal deaths. To contextualize the number of institutional maternal deaths and associated MMR, we included the institutional delivery rate and the percentage of institutional deliveries with a *major direct obstetric* complication. Both indicators were derived from EmONC assessment data. The final column includes the 2015 population-based MMR estimated by the Maternal Mortality Estimation Inter-Agency Group [7], also included for context, although most assessments occurred before 2015. Institutional delivery rates ranged from 7% in Ethiopia in 2008–9 to 113% in Mongolia (likely explained by a non-standard sampling strategy of 21 hospitals and their catchment areas). The next highest institutional delivery rate was 88% in São Tomé & Príncipe. Institutional MMRs ranged from 2130 maternal deaths per 100,000 deliveries in Chad to 32 in Mongolia.

Countries with relatively low coverage of institutional deliveries such as Haiti, Niger, Sierra Leone, Ethiopia, South Sudan, Angola, and Chad tended to have high institutional MMRs, suggesting that a disproportionate number of women delivering in facilities experienced serious complications. To some extent, the percentage of deliveries with major obstetric complications supports this pattern where high percentages were found in countries with high institutional MMRs. However, countries such as the Democratic Republic of Congo or Afghanistan also exhibited relatively low institutional delivery rates, 10% or more of deliveries with complications, and had institutional MMRs of less than 200, making it difficult to discern any robust pattern. A high percentage of complicated deliveries could also reflect the type of facility surveyed, e.g. Ecuador (30%) and Mongolia (28%), where only hospitals were assessed.

### Causes of institutional maternal deaths and cause specific case fatality rates

**Fig. 1:**
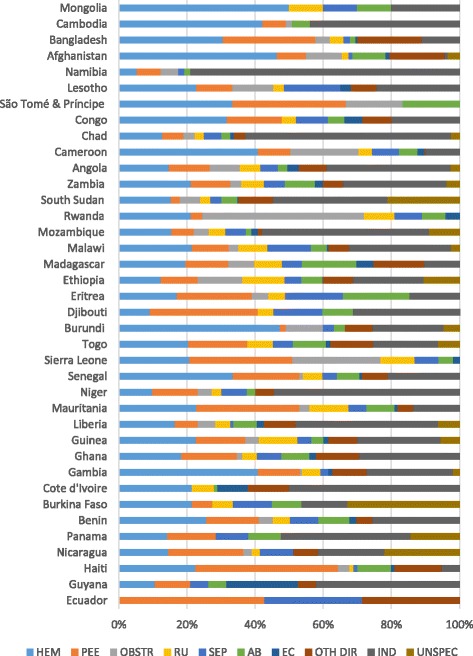
Distribution of causes of maternal death (38 countries). São Tomé & Príncipe, Sierra Leone, Rwanda and Ecuador reported only direct causes of maternal death; HEM=hemmorrhage; PEE=pre-eclampsia, eclampsia; OBSTR=obstructed/prolonged labor; RU=ruptured uterus; SEP=sepsis; AB=abortion; EC=ectopic pregnancy; OTH DIR=other direct causes of death; IND=indirect causes of death; UNSPEC=unspecified/unknown cause of death

Figure 1 shows the distribution of all reported causes of maternal death for 38 countries. In 20 countries, hemorrhage and hypertensive diseases (severe pre-eclampsia/eclampsia) approached or exceeded 40% of maternal deaths. Similarly, 10 countries reported similar levels of indirect causes of maternal death.

Only 33 countries reported the number of women with major obstetric complications by type of complication, found in the regional summaries of Table 2 (upper panel). In the Latin America and Caribbean region, hypertensive disorders ranked first (41% of direct maternal deaths), while hemorrhage ranked first in sub-Saharan Africa (33%) and Asia (42%). The lower panel shows that in sub-Saharan Africa 61% of maternal deaths were attributable to direct causes, 35% to indirect causes and 4% were unspecified or unknown, while Latin America and the Caribbean and Asian regions were weighted towards a larger proportion of direct causes of death.

**Table 2:** Numeric and percent distribution of direct obstetric complications and all maternal deaths by cause and region (33 countries)

	All regions					Latin America & the Caribbean^a^					Sub-Saharan Africa^b^					Asia^c^				
	Complications		Deaths		CFR	Complications		Deaths		CFR	Complications		Deaths			Complications		Deaths		CFR
	*n*	%	*n*	%		*n*	%	*n*	%		*n*	%	*n*	%	CFR	*n*	%	*n*	%	
Direct complications/causes																				
Hemorrhage	183,232	26%	2902	33%	1.6%	5202	11%	50	23%	1.0%	149,093	28%	2588	33%	1.7%	28,937	26%	264	42%	0.9%
Hypertensive disease	71,658	10%	1689	19%	2.4%	9441	19%	90	41%	1.0%	47,993	9%	1470	19%	3.1%	14,224	13%	129	21%	0.9%
Abortion	65,594	9%	653	8%	1.0%	3837	8%	20	9%	0.5%	42,548	8%	598	8%	1.4%	19,209	17%	35	6%	0.2%
Postpartum sepsis	16,363	2%	815	9%	5.0%	969	2%	11	5%	1.1%	13,994	3%	793	10%	5.7%	1400	1%	11	2%	0.8%
Obstr/prolong labor	154,648	22%	773	9%	0.5%	6037	12%	7	3%	0.1%	128,099	24%	722	9%	0.6%	20,512	18%	44	7%	0.2%
Ectopic pregnancy	13,732	2%	211	2%	1.5%	727	1%	6	3%	0.8%	10,756	2%	200	3%	1.9%	2249	2%	5	1%	0.2%
Ruptured uterus	8727	1%	719	8%	8.2%	122	0.2%	3	1%	2.5%	7786	1%	695	9%	8.9%	819	1%	21	3%	2.6%
Other	185,241	26%	929	11%	0.5%	22,639	46%	30	14%	0.1%	136,907	25%	786	10%	0.6%	25,695	23%	113	18%	0.4%
Total	699,195	100%	8691	100%	1.2%	48,974	100%	217	100%	0.4%	537,176	100%	7852	100%	1.5%	113,045	100%	622	100%	0.6%
Direct causes			8691	63%				217	82%				7852	61%				622	89%	
Indirect causes			4660	34%				33	13%				4556	35%				71	10%	
Unspecified causes			539	4%				12	5%				518	4%				9	1%	
TOTAL			13,890	100%				262	100%				12,926	100%				702	100%	

Note: *CFR* Case fatality rate; “other” direct complications included premature rupture of membranes, malpresentation, preterm labor, post-term labor, previous cesarean delivery, cord prolapse, multiple gestations and others. “Other” direct deaths include embolism, anesthesia complications, and others

^a^Includes Ecuador, Guyana, Haiti, Nicaragua and Panama

^b^Includes Benin, Gambia, Ghana, Guinea, Liberia, Mauritania, Niger, Senegal, Togo, Burundi, Djibouti, Eritrea, Ethiopia, Malawi, Mozambique, South Sudan, Zambia, Angola, Cameroon, Chad, Congo, São Tomé & Príncipe, Lesotho and Namibia

^c^Includes Afghanistan, Bangladesh, Cambodia and Mongolia

Ruptured uterus had the highest cause-specific case fatality rate in each region, ranging from 8.9% in sub-Saharan Africa to 2.5% in the Latin America and Caribbean region. In other words, for every 100 women with a ruptured uterus in sub-Saharan Africa, 9 will die. The second highest specific cause of death was postpartum sepsis in sub-Saharan Africa (5.7%) and in Latin America and the Caribbean (1.1%), while in Asia, hypertensive diseases and hemorrhage tied for second (0.9%).

Seventeen sub-Saharan African countries reported the number of indirect complications and deaths due to malaria in pregnancy, HIV/AIDS, severe anemia and other indirect causes of death. A few countries reported sickle cell anemia, hepatitis and diabetes, but most countries placed these women in the category of “other” indirect complications; 68% of indirect complications were malaria-related, 13% to HIV/AIDS, 7% to anemia, and 12% to “other indirect” complications. Less than 1% of indirect complications reported were cases of sickle cell anemia, hepatitis or diabetes, but more than a third of pregnant or recently delivered women with hepatitis or diabetes died before discharge (underreporting of survivors was likely). The case fatality rate for anemia was 2.3%, 1.0% for HIV/AIDS, 0.5% for malaria, and 1.6% for “other” indirect complications (data not shown).

**Fig. 2:**
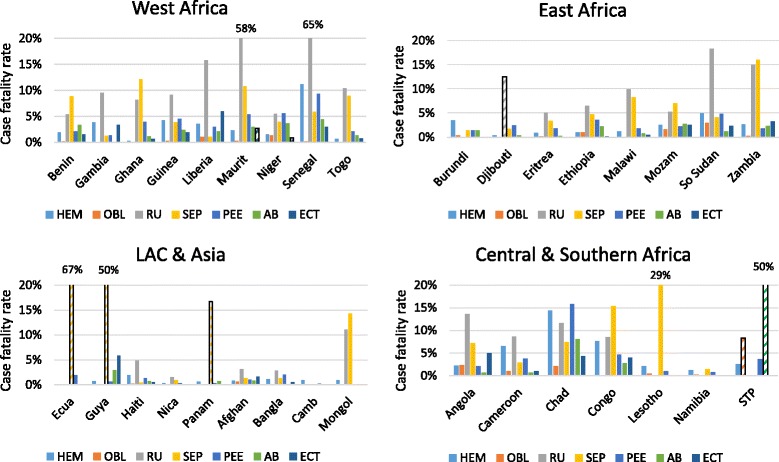
Cause-specific case fatality rates by region and country (33 countries). Hashed bars represent rates based on very small numbers; HEM=hemmorrhage; OBL=obstructed/prolonged labor; RU=ruptured uterus; SEP=sepsis; PEE=pre-eclampsia, eclampsia; AB=abortion; ECT=ectopic pregnancy; LAC=Latin America & the Caribbean; Maurit=Mauritania; Mozam=Mozambique; Ecua=Ecuador; Guya=Guyana; Nica=Nicaragua; Panam=Panama; Afghan=Afghanistan; Bangla=Bangladesh; Camb=Cambodia; Mongol=Mongolia; STP=São Tomé e Príncipe

Figure 2 depicts the cause-specific case fatality rates for direct obstetric complications, organized by countries within regions. Hashed bars indicate a case fatality rate based on small numbers. Angola, Chad, Congo Brazzaville, Guinea, Mauritania, Senegal, and South Sudan experienced high rates (≥4%) across three or more complications.

### Institutional stillbirth and pre-discharge early neonatal mortality rates

**Fig. 3:**
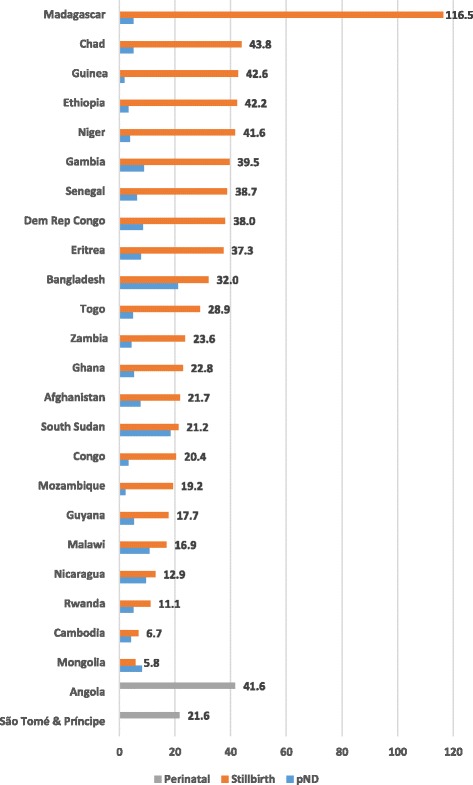
Institutional stillbirth and pre-discharge neonatal death (pND) rates (25 countries)

Twenty-three countries calculated stillbirth and pre-discharge early neonatal death rates, nine of which did not distinguish between antepartum and intrapartum stillbirths, while two countries calculated only a pre-discharge perinatal mortality rate (Table 3 and Fig. 3). Stillbirth rates ranged from 5.8 per 1000 deliveries in Mongolia, to 116.5 in Madagascar. Pre-discharge neonatal death rates were often much smaller than the stillbirth rates except for Mongolia. Early neonatal death rates ranged from 1.8 in Guinea to 21 in Bangladesh. The ratio of stillbirths to early neonatal deaths varied widely across countries, ranging from the outlier ratios of 26 and 25 stillbirths to 1 pre-discharge neonatal death in Madagascar and Guinea to 0.7 to 1 in Mongolia. Mongolia had the lowest institutional and population-based MMR and the lowest stillbirth rate, but its pre-discharge early neonatal death rate was similar to that of many countries, and begs for an explanation – a question of sampling or quality of newborn care?

**Table 3:** Institutional stillbirth and pre-discharge early neonatal mortality rates (23 countries)

Region, country and year of data collection	Institu-tional deliveries	Ante-partum SBs	Intra-partum SBs	Unspe-cified SBs	Total SBs	SB rate per 1000 deliveries	pNDs	pND rate per 1000 live births	SB:pND ratio
LAC									
Guyana 2010	12,803	70	67	89	226	17.7	65	5.2	3.5
Nicaragua 2006	94,136	NR	NR	NR	1210	12.9	889	9.6	1.4
Western Africa									
Gambia 2012	51,518	1023	944	66	2033	39.5	433	8.8	4.7
Ghana 2010	434,508	3989	4685	1223	9897	22.8	2201	5.2	4.5
Guinea 2011	141,724	1944	1457	3639	6040	42.6	242	1.8	25.0
Niger 2010	152,415	1171	4105	1072	6348	41.6	545	3.7	11.6
Senegal 2013	237,494	3761	3345	2078	9184	38.7	1439	6.3	6.4
Togo 2012	133,119	974	1728	1150	3852	28.9	634	4.9	6.1
Eastern Africa									
Eritrea 2008	25,000	NR	NR	NR	933	37.3	185	7.7	5.0
Ethiopia 2008–9	174,561	NR	NR	NR	7366	42.2	522	3.1	14.1
Madagascar 2009	118,774	NR	NR	NR	13,832	116.5	527	5.0	26.2
Malawi 2014	476,272	3632	4403	NR	8035	16.9	5028	10.7	1.6
Mozambique 2012^a^	647,944	828	3440	8200	12,468	19.2	1380	2.2	9.0
Rwanda 2007^a^	207,738	17,456	5618	NR	23,074	11.1	9432	5.1	2.4
South Sudan 2013	52,842	208	541	373	1122	21.2	948	18.3	1.2
Zambia 2014–15	475,646	NR	NR	NR	11,233	23.6	1980	4.3	5.7
Central Africa									
Chad 2011	49,202	274	814	NR	2155	43.8	239	5.1	9.0
Congo 2012	85,038	657	856	219	1732	20.4	264	3.2	6.6
Dem Rep Congo 2011	156,546	NR	NR	NR	5949	38.0	1271	8.4	4.7
Asia									
Afghanistan 2009	192,627	NR	NR	NR	4177	21.7	1422	7.5	2.9
Bangladesh 2012	253,728	NR	NR	NR	8119	32.0	5158	21.0	1.6
Cambodia 2014	119,931	92	715	NR	807	6.7	479	4.0	1.7
Mongolia 2009	30,131	NR	NR	NR	175	5.8	242	8.1	0.7

*NR* not reported, *SB* stillbirth, *pND* pre-discharge early neonatal death, dying before discharge or within the first 24 h, whichever came first

^a^Mozambique and Rwanda adjusted to reflect 12 months of information

## Discussion

This institutional assessment compiles data from many countries where every country set out with similar objectives, used a similar methodology and data collection instrument, and had common indicators. By design, each assessment captured a complete recording of all maternal deaths by cause and common perinatal outcomes. This is a strength that other multi-country studies have not shared. In this overview, we gathered service statistics from more than 15,400 health care facilities that mirror findings from more complex modeling exercises, regardless of differences in methodology and reference populations.

We saw hypertensive diseases as the predominant cause of institutional maternal death in the Latin America and Caribbean region and hemorrhage highlighted in Asian countries, despite the small number of surveys in each of those regions. Meanwhile, hemorrhage was the predominant cause of institutional maternal deaths in sub-Saharan African countries, a region also distinguished by its large proportion of indirect maternal deaths.

In Table 4 below we compare the overall distribution of institutional causes of 14,785 deaths from 26 sub-Saharan African countries with the population-based distribution found in the WHO 2003–2009 systematic review (in both cases, unknown causes of death were excluded). Cases of ruptured uterus and obstructed labor were included in “other direct causes” for the EmONC Assessments while these cases were likely assigned to hemorrhage or sepsis in the WHO study [14]. The degree of similitude in the distribution of causes is both validating and reassuring but may also point to possible data quality issues and/or differences between *all* deaths versus just those occurring in facilities.

**Table 4:** Comparison of causes of maternal mortality in sub-Saharan countries by different sources

For sub-Saharan African countries	EmONC Assessments (institution-based)	WHO 2003–2009 Review (population-based)
Hemorrhage	21.0%	24.5%
Abortion + ectopic pregnancy	7.2%	9.6%
Sepsis	6.0%	10.3%
Hypertensive diseases	10.5%	16.0%
Other direct causes	17.8%	11.1%
Indirect causes	37.5%	28.6%

The larger proportion of indirect causes found in this paper is noteworthy but it also may be underreported especially where comorbidities were common. During the training, data collectors were instructed to classify a maternal death as direct if there was evidence of both direct and indirect causes. For programmatic purposes, indirect causes of maternal mortality require a greater focus of attention, not just for purposes of reporting but also for health service delivery organization to intervene early to prevent these deaths.

We also observed that institutional stillbirth rates tended to be substantially higher than early neonatal death rates, and that countries with high institutional stillbirth rates also tended to exhibit high institutional MMRs. According to other studies, we might have expected the ratio of stillbirths to early neonatal deaths to be approximately 1.3 to 1, but these institutional data suggest a lower ratio, i.e., more stillbirths than expected [20, 21]. Unfortunately, given the uneven reporting of whether the stillbirth was macerated or intrapartum, the often-cited ratio of 1 intrapartum stillbirth to 3 macerated stillbirths could not be assessed [22].

The 2030 ENAP target for the stillbirth rate is 12/1000 total births and the target neonatal death rate is the same, but among live births. At this time, most countries in this overview are far from reaching the stillbirth target and many countries would fail to reach the neonatal target of 12, although this is more difficult to ascertain given the censoring of data since so many women and their newborns are discharged within 12 h of delivery. A recent six-country study of early neonatal mortality showed that neonatal deaths in the first six and 24 h account for one-third and 46%, respectively, of all neonatal deaths [23]. Therefore, a doubling or tripling of the early neonatal deaths observed in this overview might provide a rough estimate of the actual neonatal death rate. But like the MMR, it is unclear whether institutional rates and ratios are likely to be higher or lower than the population-based rates. Nevertheless, high stillbirth rates observed in the EmONC assessments give pause; the global stillbirth rate for 2015 was 18.4 per 1000 births, while the rate for sub-Saharan Africa was 28.7 [4]. According to the authors of recent trend data for stillbirth rates, when compared to high quality vital registration data, facility data tend to overestimate the stillbirth rate due to selection bias [4, 5].

Despite evidence for reductions in maternal and perinatal mortality over the last two decades, this multi-country overview leads to recommendations for clinical practice and policy if we are to move towards the goal of ending preventable maternal and newborn deaths. From the clinical perspective, although fewer in absolute numbers than hemorrhage or hypertensive disorders, uterine rupture and maternal sepsis were the most lethal complications. The literature consistently shows the elevated risk of mortality from ruptured uterus [24–27]. High case fatality rates for uterine rupture suggest poor diagnostic skills, inadequate patient monitoring after admission and delays in appropriate treatment [28], or perhaps inappropriate or overuse of augmentation or induction. Several studies point to high rates of rupture after admission [25, 29]. Ruptured uterus is also an indication that women with obstructed labor or at risk of rupture, e.g. having a previous uterine scar, still experience difficulties in accessing surgical care in a timely manner.

Considerable international investment has focused on reducing deaths due to hemorrhage and hypertensive disorders, given how many deaths are attributable to these complications. Both have well-known pharmacological solutions as well as effective preventative measures with active management of the third stage of labor and the potential to detect high blood pressure and proteinuria during antenatal care. Ruptured uterus might be viewed as requiring more complex multi-sectoral fixes – improved road networks, better communication and transportation options, as well as the human resources who can and will monitor the progression of labor, follow protocol, and perform cesarean delivery. Sepsis may require more of a professional culture change towards infection prevention, more accessible water, sanitation and hygiene infrastructure as well as antenatal screening.

To optimize the investment of an EmONC assessment, it should be followed by multilevel planning and implementation phases. In 2016, only six of the countries mentioned in this publication have set up such processes that include maternal and newborn care monitoring in EmONC facilities (Burkina Faso, Cambodia, Haiti, Madagascar, Niger and Togo). However, this number is likely to increase significantly in 2017. The production, analysis and utilization of data by providers with the support of coaches also contribute to improve quality of care.

### Limitations

Without access to the original data, we could not standardize reporting nor could we stratify by level of facility or management authority, which would have allowed a deeper understanding of which deaths occurred where and how many. There may also have been bias in how causes of death were ascertained across countries although training guidelines were the same across most countries. It is possible that some countries were more comfortable than others using ICD-MM. Going forward, EmONC assessments should better align the cause of death categories with ICD-MM, thus making these data more attractive as an additional source for global estimates.

Systematic documentation of stillbirths is at an early stage in many low and middle income countries and the differentiation between antepartum and intrapartum stillbirths is not yet standard practice across or within countries. Like maternal deaths, stillbirth rates and early neonatal death rates are susceptible to errors of omission and misclassification [30]. Especially critical may be widespread misclassification of early neonatal deaths as intrapartum stillbirths due to lack of diagnostic skill, environmental pressure or convenience. Countries such as Madagascar, Guinea and Ethiopia that exhibited an extreme ratio of stillbirths to early neonatal deaths should investigate these rates to understand possible contributory clinical and reporting practices. Caregivers need access to simple equipment to measure the presence of fetal heart beats on admission, training to make accurate assessments and the paper or electronic tools that encourage reporting whether the fetal death was antepartum or intrapartum [31]. As long as large numbers of stillbirths and birth weights remain unspecified, the use of the intrapartum and early neonatal death rate as an indicator for quality of intrapartum care will be compromised or relegated to the status of a special study.

The recording of maternal deaths is likely to be incomplete given the primary sources of the statistics – routine paper-based logbooks – the extended coverage of 12 months, and for unintentional and intentional reasons. Obstetric complications are also likely to be undercounted as they are rarely collected by routine health management information systems. Specific case fatality rates suggest inconsistent reporting and recording across facilities and countries. For example, the case fatality rate of 1% for HIV in sub-Saharan Africa was surprisingly low as were the case fatality rates of 0% for hemorrhage in Ecuador, and 0% for obstructed labor in Ghana, Togo and the Gambia. Nevertheless, by supporting the EmONC assessments we have learned that registers and logbooks tend to be more complete than facility reports of aggregated data. We also observed that where maternal death surveillance and response (MDSR) initiatives were well entrenched, the quality of the maternal death data in the EmONC assessments appeared to be of higher quality than where MDSR efforts were in their early stages. As countries adopt *Making Every Baby Count: Audit and Review of Stillbirths and Neonatal Deaths,* routine data on newborn outcomes are likely to improve in quality as will our understanding of why deaths occur and how to intervene.

## Conclusions

As skilled delivery coverage increases and maternal mortality declines, women who die in facilities may no longer represent the tip of an iceberg, but most maternal deaths. With appropriate reflection, institutional stillbirth and early neonatal death rates, causes of maternal death and case fatality rates can guide management on how to improve health workers’ capacity to meet the demand for emergency care, including record-keeping, and identify hotspots of where and what is needed to reduce delays in seeking, reaching and receiving care. Facility-level data will become all the more important and thus efforts to improve data quality are crucial.

## Abbreviations

AMDD: Averting Maternal Death and Disability
EmONC: emergency obstetric and newborn care
ENAP: Every Newborn Action Plan
ICD-10: International statistical classification of diseases and related health problems, 10th edition
ICD-MM: The WHO application of ICD-10 to deaths during pregnancy, childbirth, and the puerperium (maternal mortality)
MDSR: Maternal death surveillance and response
MMR: Maternal mortality ratio
SDG: Sustainable Development Goal
UNFPA: United Nations Population Fund
UNICEF: United Nations Children’s Fund
WHO: World Health Organization

## References

[1] Moxon SG, Ruysen H, Kerber KJ, Amouzou A, Fournier S, Grove J, Moran AC, Vaz LM, Blencowe H, Conroy N (2015). Count every newborn; a measurement improvement roadmap for coverage data. BMC Pregnancy Childbirth.

[2] World Health Organization: Every Newborn: an Action Plan to End Preventable Deaths (ENAP) [http://www.who.int/maternal_child_adolescent/topics/newborn/en/]. Accessed 7 Sept 2017.

[3] World Health Organization: Strategies toward Ending Preventable Maternal Mortality (EPMM) [http://who.int/reproductivehealth/topics/maternal_perinatal/epmm/en/]. Accessed 15 May 2017.

[4] Blencowe H, Cousens S, Jassir FB, Say L, Chou D, Mathers C, Hogan D, Shiekh S, Qureshi ZU, You D (2016). National, regional, and worldwide estimates of stillbirth rates in 2015, with trends from 2000: a systematic analysis. Lancet Glob Health.

[5] Cousens S, Blencowe H, Stanton C, Chou D, Ahmed S, Steinhardt L, Creanga AA, Tuncalp O, Balsara ZP, Gupta S (2011). National, regional, and worldwide estimates of stillbirth rates in 2009 with trends since 1995: a systematic analysis. Lancet.

[6] Wang H, Liddell CA, Coates MM, Mooney MD, Levitz CE, Schumacher AE, Apfel H, Iannarone M, Phillips B, Lofgren KT (2014). Global, regional, and national levels of neonatal, infant, and under-5 mortality during 1990-2013: a systematic analysis for the global burden of disease study 2013. Lancet.

[7] World Health Organization, UNICEF, UNFPA, World Bank Group, United Nations Population Divsion: Trends in Maternal Mortality: 1990 to 2015. In*.* Geneva, Switzerland; 2015:38.

[8] Mgawadere F, Kana T, van den Broek N (2017). Measuring maternal mortality: a systematic review of methods used to obtain estimates of the maternal mortality ratio (MMR) in low- and middle-income countries. Br Med Bull.

[9] AbouZahr C, Wardlaw T, Hill K (2004). Maternal mortality in 2000: estimates developed by WHO.

[10] World Health Organization, UNICEF, UNFPA, The World Bank, United Nations Population Divsion: Trends in maternal mortality: 1990 to 2013. Estimates by WHO, UNICEF, UNFPA, The World Bank and the United Nations Population Division. In*.* Geneva, Switzerland; 2014: 56.

[11] Kassebaum NJ, Bertozzi-Villa A, Coggeshall MS, Shackelford KA, Steiner C, Heuton KR, Gonzalez-Medina D, Barber R, Huynh C, Dicker D (2014). Global, regional, and national levels and causes of maternal mortality during 1990-2013: a systematic analysis for the global burden of disease study 2013. Lancet.

[12] Kassebaum NJ, Collaborators GMM (2016). Global, regional, and national levels of maternal mortality, 1990-2015: a systematic analysis for the global burden of disease study 2015. Lancet.

[13] Khan KS, Wojdyla D, Say L, Gulmezoglu AM, Van Look PFA (2006). WHO analysis of causes of maternal death: a systematic review. Lancet.

[14] Say L, Chou D, Gemmill A, Tunçalp O, Moller A, Daniels J, Gulmezoglu A, Temmerman M, Alkema L (2014). Global causes of maternal death: a WHO systematic analysis. Lancet Global Health..

[15] United Nations: The millennium development goals report 2015 [http://www.un.org/millenniumgoals/2015_MDG_Report/pdf/]. Accessed 21 May 2017.

[16] Keyes EB, Haile-Mariam A, Tesfaye NB, Wasihun GA, Pearson L, Abdullah M, Kebede H (2011). Ethiopia’s Assessment of emergency obstetric and newborn care: setting the gold standard for national facility-based assessments. Int J Gynecol Obstet.

[17] Bailey P, van Roosmalen J, Mola G, Evans C, de Bernis L, Dao B (2017). Assisted vaginal delivery in low and middle income countries: an overview. B J O G.

[18] Averting Maternal Death and Disability (AMDD) Toolkit [https://www.mailman.columbia.edu/research/averting-maternal-death-and-disability-amdd/toolkit]. Accessed 21 May 2017.

[19] Fauveau V. New indicator of quality of emergency obstetric and newborn care. Lancet. 2007;370(9595):1310–0. 10.1016/S0140-6736(07)61571-2 17933644

[20] WHO: The World Health Report: 2005 (2005). Make every mother and child count.

[21] Engmann C, Matendo R, Kinoshita R, Ditekemena J, Moore J, Goldenberg RL, Tshefu A, Carlo WA, McClure EM, Bose C (2009). Stillbirth and early neonatal mortality in rural Central Africa. Int J Gynaecol Obstet.

[22] Lawn J, Shibuya K, Stein C (2005). No cry at birth: global estimates of intrapartum stillbirths and intrapartum-related neonatal deaths. Bull World Health Organ.

[23] Baqui AH, Mitra DK, Begum N, Hurt L, Soremekun S, Edmond K (2016). Neonatal mortality within 24 hours of birth in six low- and lower-middle-income countries. Bull World Health Organ.

[24] Hofmeyr GJ, Say L, Gulmezoglu AM (2005). WHO systematic review of maternal mortality and morbidity: the prevalence of uterine rupture. BJOG.

[25] van den Akker T, Mwagomba B, Irlam J, van Roosmalen J (2009). Using audits to reduce the incidence of uterine rupture in a Malawian district hospital. Int J Gynecol Obstet.

[26] Nakimuli A, Nakubulwa S, Kakaire O, Osinde MO, Mbalinda SN, Nabirye RC, Kakande N, Kaye DK (2016). Maternal near misses from two referral hospitals in Uganda: a prospective cohort study on incidence, determinants and prognostic factors. BMC Pregnancy Childbirth.

[27] Nyamtema AS, Mwakatundu N, Dominico S, Mohamed H, Pemba S, Rumanyika R, Kairuki C, Kassiga I, Shayo A, Issa O (2016). Enhancing maternal and Perinatal health in under-served remote areas in sub-Saharan Africa: a Tanzanian model. PLoS One.

[28] Miller S, Abalos E, Chamillard M, Ciapponi A, Colaci D, Comande D, Diaz V, Geller S, Hanson C, Langer A (2016). Beyond too little, too late and too much, too soon: a pathway towards evidence-based, respectful maternity care worldwide. Lancet.

[29] Mbaruku G, van Roosmalen J, Kimondo I, Bilango F, Bergstrom S (2009). Perinatal audit using the 3-delays model in western Tanzania. Int J Gynaecol Obstet.

[30] Maaloe N, Housseine N, Bygbjerg IC, Meguid T, Khamis RS, Mohamed AG, Nielsen BB, van Roosmalen J (2016). Stillbirths and quality of care during labour at the low resource referral hospital of Zanzibar: a case-control study. BMC Pregnancy Childbirth.

[31] Goldenberg RL, McClure EM, Kodkany B, Wembodinga G, Pasha O, Esamai F, Tshefu A, Patel A, Mabaye H, Goudar S (2013). A multi-country study of the “intrapartum stillbirth and early neonatal death indicator” in hospitals in low-resource settings. Int J Gynecol Obstet.

